# *Dendranthema boreale* (Makino) Ling ex Kitam. Flower Extract Ameliorates Oxidative Stress-Induced Cellular Damage in HaCaT Keratinocytes by Regulating MAPK Signaling

**DOI:** 10.4014/jmb.2505.05005

**Published:** 2025-07-18

**Authors:** You Kyeong Lee, Parkyong Song, Seo Young Choi, Mi Song Shin, Ji Sun Hwang, Hong-Joo Son, Yu-Jin Kim, Wanil Kim, Kwang Min Lee

**Affiliations:** 1 Department of Life Science and Environmental Biochemistry, Pusan National University, Miryang 50463, Republic of Korea; 2 Department of Convergence Medicine, Pusan National University School of Medicine, Yangsan 50612, Republic of Korea; 3 New Drug Development Center, Daegu-Gyeongbuk Medical Innovation Foundation, Daegu 41061, Republic of Korea; 4 Department of Biochemistry, Department of Convergence Medical Science, and Institute of Medical Science, Gyeongsang National University College of Medicine, Jinju 52727, Republic of Korea

**Keywords:** *Dendranthema boreale* (Makino) Ling ex Kitam. flower, hydrogen peroxide, oxidative stress, HaCaT Keratinocytes, skin damage

## Abstract

Oxidative stress plays a critical role in skin aging and in various dermatological disorders by promoting inflammation, apoptosis, and cellular dysfunction. Among reactive oxygen species (ROS), hydrogen peroxide (H_2_O_2_) readily penetrates cell membranes, triggering oxidative damage. This study investigated the protective effects of the *Dendranthema boreale* (Makino) Ling ex Kitam. flower extract (DBE) against H_2_O_2_-induced oxidative stress in HaCaT keratinocytes and explored the underlying molecular mechanisms. DBE (30-80 μg/ml) significantly attenuated H_2_O_2_-induced cytotoxicity by reducing cleaved caspase-3 activation and lowering the Bax/Bcl-2 ratio, thereby inhibiting apoptosis. Furthermore, DBE selectively suppressed JNK and ERK phosphorylation while having no effect on p38 MAPK activation. Inflammatory responses were also modulated, as DBE inhibited NF-κB p65 phosphorylation and downregulated COX-2 expression, a key mediator of oxidative stress-induced inflammation. These findings indicate that DBE protects HaCaT keratinocytes from oxidative stress-induced cellular damage by promoting cell survival, suppressing apoptosis, and modulating the key signaling pathways involved in oxidative stress and inflammation. This study provides foundational insights into the potential therapeutic and cosmetic applications of DBE for the prevention of oxidative stress-related skin disorders.

## Introduction

The skin, the body's largest organ, acts as a protective shield against external factors, such as ultraviolet (UV) radiation, harmful chemicals, microorganisms, and environmental stimuli [1]. These external challenges trigger the production of reactive oxygen species (ROS), which play a vital role in supporting normal physiological processes at low concentrations [2]. However, oxidative stress occurs when their levels exceed the detoxifying capacity of cellular antioxidant enzymes [3, 4].

Oxidative stress plays a critical role in skin aging, inflammation, and disorders, such as atopic dermatitis and psoriasis [5]. Among ROS, hydrogen peroxide (H_2_O_2_) is particularly important due to its ability to readily cross cellular membranes, where it initiates free radical generation and lipid peroxidation [6-8]. These processes inhibit cell proliferation, accelerate cellular senescence, and induce cell death, contributing to tissue damage and disease progression [9]. Due to its stability, accessibility, and ability to simulate oxidative damage in cellular systems, H_2_O_2_ is widely used as a model agent for *in vitro* studies, enabling researchers to investigate protective mechanisms and develop therapeutic and cosmetic strategies for mitigating oxidative stress [10, 11].

*Dendranthema boreale* (Makino) Ling ex Kitam. is a perennial herbaceous plant belonging to the Asteraceae family, which grows predominantly in East Asian regions, including Korea, China, and Japan [12-14]. Known for its bioactive constituents, such as polyacetylenes, essential oils, and flavonoids, this plant has been traditionally used for its anti-inflammatory, detoxifying, and antioxidant properties, making it a valuable resource in cosmetics, food products, and traditional medicine [12, 15-17]. However, despite its diverse medicinal applications, there is no scientific evidence supporting its role as an anti-skin-damage agent, nor have there been any reports on its protective effects against H_2_O_2_-induced oxidative stress in the context of skin damage. Therefore, this study aimed to explore the protective effects of DBE on HaCaT keratinocytes exposed to H_2_O_2_, with the goal of providing foundational insights into the development of therapeutic and cosmetic applications targeting oxidative stress-induced skin damage.

## Materials and Methods

### Cell Culture

HaCaT human epithelial keratinocyte cells were obtained from AddexBio (USA). Cells were cultured in Dulbecco's Modified Eagle Medium (DMEM; Welgene Inc., Republic of Korea) supplemented with 10% fetal bovine serum (FBS; Gibco, USA) and 1% antibiotics (50 μg/ml streptomycin and 50 U/ml penicillin). The incubation conditions were maintained at 37°C with 5% CO_2_. To evaluate the effects of DBE, HaCaT keratinocytes were seeded in 12-well plates and allowed to reach 80% confluence. Subsequently, the cells were pretreated with different concentrations of DBE (30, 50, and 80 μg/ml) for 24 h. After pretreatment, the medium was discarded and the cells were washed twice with phosphate-buffered saline (PBS) before exposure to 1 mM H_2_O_2_ for 12 h.

### Preparation of *Dendranthema boreale* (Makino) Ling ex Kitam. Flower Extract

The plant extract powder (code number: KPM038-056) used in this study was obtained from the Korea Plant Extract Bank (KPEB; https://portal.kribb.re.kr/kpeb) of the Korea Research Institute of Bioscience and Biotechnology (KRIBB, Republic of Korea). Briefly, dried whole portions of *Dendranthema boreale* (Makino) Ling ex Kitam. flowers were cut into small pieces and extracted with 99.9% methanol for 3 d. After extraction, the solutions were filtered, concentrated, and dried to obtain a powder. The powdered extract was dissolved in dimethyl sulfoxide prior to use.

### Cell Viability Assay

Cell viability was assessed using a water-soluble tetrazolium salt 1 (WST-1) assay (EZ-CyTox, Republic of Korea) following the manufacturer’s instructions. HaCaT keratinocytes were seeded into 96-well plates at a density of 2.5 × 10^4^ cells/well and treated with the indicated concentrations of either H_2_O_2_ or DBE after being washed twice with PBS. The optical density of control cells was set to 100%.

A Trypan blue exclusion assay was conducted to evaluate cell viability according to the manufacturer’s protocol. HaCaT keratinocytes were seeded into 12-well plates and treated with either H_2_O_2_ or DBE (80 μg/ml). Following treatment, an aliquot of the cell suspension was centrifuged, and the supernatant was removed. The cell pellet was resuspended in serum-free complete medium, diluted in PBS, and mixed with 0.4% Trypan blue solution at a 1:1 ratio. A drop of this mixture was placed on a hemocytometer, and both viable (unstained) and non-viable (stained) cells were counted separately under a microscope:

Viable cells (%) = (total number of viable cells/total number of cells) × 100

### Immunoblot Analysis

Proteins extracted from HaCaT keratinocytes were separated using sodium dodecyl sulfate-polyacrylamide gel electrophoresis (SDS-PAGE) and subsequently transferred onto nitrocellulose membranes. To prevent nonspecific binding, the membranes were blocked with 3% bovine serum albumin in a TBS-T buffer (137 mM NaCl, 20 mM Tris-Cl, pH = 7.6, and 0.1% Tween 20). Following blocking, the membranes were incubated with primary antibodies targeting various proteins, including cleaved caspase-3 (Cell Signaling Technology, 9664), Bax (CST, 2772), Bcl-2 (CST, 15071), p44/42 MAPK (ERK1/2; R&D Systems, MAB1576), phospho-p44/42 MAPK (ERK1/2; CST, 4377), p38 MAPK (CST, 8690), phospho-p38 MAPK (CST, 4511), JNK (CST, 9252), phospho-JNK (CST, 4668), NF-κB p65 (CST, 8242), phospho-NF-κB p65 (CST, 3033), COX-2 (CST, 12282), and tubulin (Sigma-Aldrich, T6199). The membranes were then incubated with anti-rabbit or anti-mouse horseradish peroxidase-conjugated secondary antibodies (Santa Cruz Biotechnology). The protein bands were detected using an enhanced chemiluminescence detection system (Bio-Rad).

### Statistical Analysis

Significant differences between groups were determined using two-tailed unpaired Student’s *t*-tests, and multiple comparisons were performed using one- or two-way repeated-measure ANOVA with Tukey’s post-hoc test. The analysis was performed using Origin 8.0 (OriginLab Corporation, USA). Data were expressed as the mean ± standard error of the mean (SEM) of at least three independent experiments, where *p* < 0.05, as indicated in the figure legends.

## Results

### No Significantly Cytotoxic Effects on HaCaT Keratinocytes

**Fig. 1:**
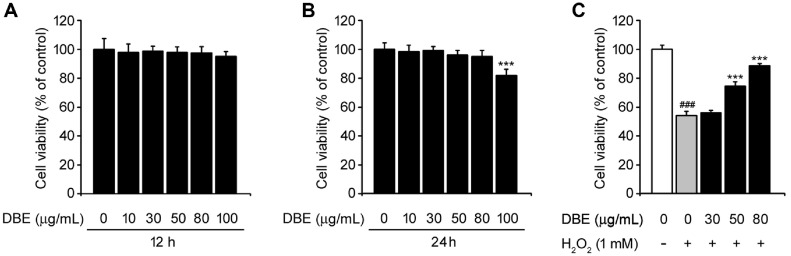
Cell viability of DBE in HaCaT keratinocytes. (**A**) HaCaT cells were exposed to DBE (0, 10, 30, 50, 80, and 100 μg/ml) for 12 h. (**B**) HaCaT cells were treated with DBE (0, 10, 30, 50, 80, and 100 μg/ml) for 24 h. (**C**) Cells were exposed to DBE for 24 h, followed by exposure to H_2_O_2_ (1 mM) for 12 h. Cell viability was detected by the WST-1 assay. The values in the bar graphs represent the mean ± SEM of at least three independent experiments. #Statistical differences (###*p* < 0.005) compared to the HaCaT control cells not treated with DBE or H_2_O_2_. *Statistical differences (*p < 0.05, **p < 0.01, ***p < 0.005) compared to cells treated with H_2_O_2_.

First, we assessed the toxicity of DBE in human keratinocyte HaCaT cells using the WST-1 assay. Various concentrations of DBE were applied for 12 and 24 h. Fig. 1A shows the cell viability after 12 h of DBE treatment, which were not significantly different up to 100 μg/ml. After 24 h, cytotoxicity was slightly observed at 100 μg/ml (Fig. 1B). Therefore, we optimized the DBE concentrations of 30, 50, and 80 μg/ml for subsequent experiments to evaluate its effect on oxidative stress in a dose-dependent manner. H_2_O_2_ is a well-known inducer of oxidative stress, which leads to cell death by activating apoptotic mediators [18-20]. To investigate the protective effects of DBE against oxidative stress, HaCaT keratinocytes were exposed to H_2_O_2_ in the presence of DBE at the respective concentrations. H_2_O_2_ treatment significantly reduced cell viability; however, pre-treatment with DBE (30-80 μg/ml) alleviated this effect in a dose-dependent manner. These results suggested that DBE mitigates H_2_O_2_-induced oxidative stress and protected HaCaT keratinocytes from oxidative damage.

### DBE Protected HaCaT Keratinocytes from Oxidative Stress-Induced Apoptosis

**Fig. 2:**
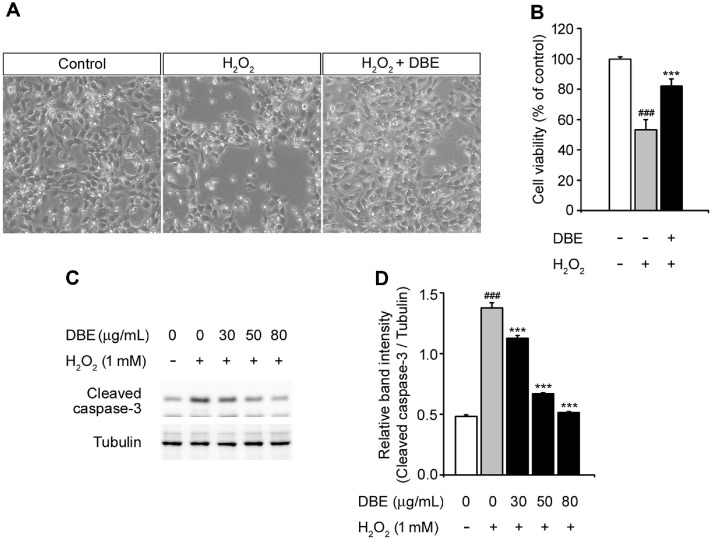
Effects of DBE on H_2_O_2_-triggered cell death in HaCaT keratinocytes. (**A**) Microscopic image of HaCaT cells with or without pretreatment with DBE and followed by exposure to H_2_O_2_. (**B**) Cell viability was evaluated using Trypan blue assay under the same conditions as (**A**). (**C**) Western blot analysis of cleaved caspase-3 expression in HaCaT cells. (**D**) The relative band intensity of cleaved caspase-3 was quantified via densitometric analysis and normalized to tubulin. The values in the bar graphs represent the mean ± SEM of at least three independent experiments. #Statistical differences (###*p* < 0.005) compared to the HaCaT control cells not treated with DBE or H_2_O_2_. *Statistical differences (*p < 0.05, **p < 0.01, ***p < 0.005) compared to cells treated with H_2_O_2_.

To investigate the effects of DBE against H_2_O_2_-stimulated morphological change, we treated HaCaT keratinocytes with H_2_O_2_ in the presence or absence of DBE [21]. Cells incubated with H_2_O_2_ exhibited shrunken and altered morphologies [22], accompanied by a marked reduction in cell viability (Fig. 2A). Using the Trypan blue exclusion assay, we not only observed cell morphology but also performed quantitative measurements. DBE treatment effectively attenuated apoptosis and significantly enhanced cell viability (Fig. 2B).

Caspase-3, a member of the cysteine-aspartate protease (caspase) family, plays a key role in apoptosis and is activated by H_2_O_2_, a major effector caspase associated with apoptosis [23-25]. Accordingly, we assessed caspase-3 activity by detecting the levels of cleaved caspase-3 protein using western blot analysis (Fig. 2C and 2D). These results demonstrate the antiapoptotic effect of DBE via the inhibition of cleaved caspase-3. When the cells were exposed to H_2_O_2_, the expression of cleaved caspase-3 increased compared to that in untreated cells. However, DBE significantly reduced cleaved caspase-3 expression in a dose-dependent manner compared with the H_2_O_2_ treatment alone. This demonstrates that DBE has the potential to protect HaCaT keratinocytes from H_2_O_2_-mediated cell death.

### DBE Decreased Bax/Bcl-2 Ratio in H_2_O_2_-Stimulated HaCaT Keratinocytes

**Fig. 3:**
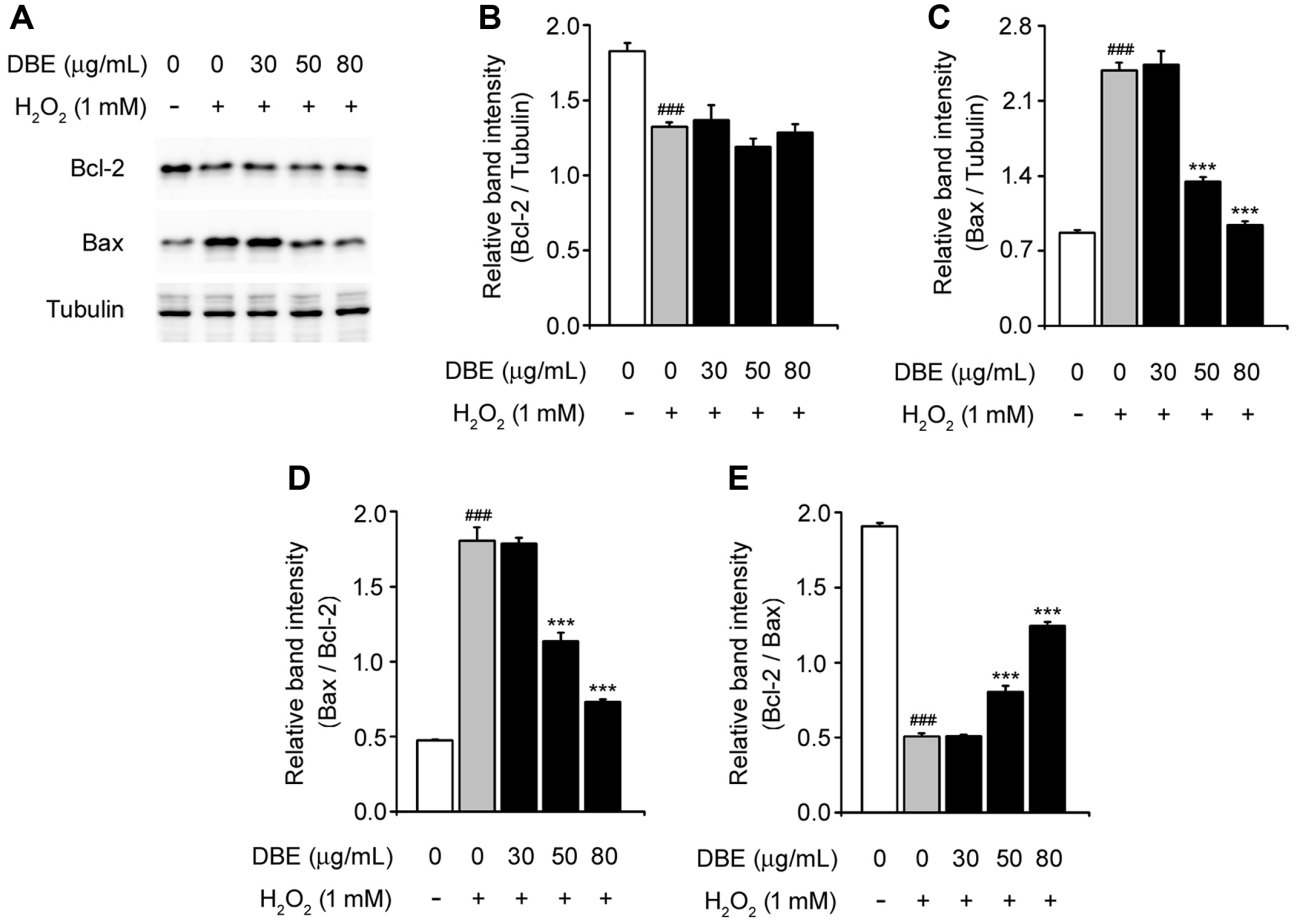
Effect of DBE on the expression of Bcl-2 and Bax in HaCaT keratinocytes exposed to hydrogen peroxide. (**A**) Western blot bands corresponding to Bcl-2 and Bax in HaCaT cells that were pre-treated with DBE and then exposed to H_2_O_2_. (**B**) Relative band intensity of Bcl-2 (quantified by densitometric analysis and normalized to that of tubulin). (**C**) Relative band intensity of Bax (quantified using densitometric analysis and normalized to tubulin). (**D**) Bax/Bcl-2. (**E**) Bcl-2/Bax ratio. The values in the bar graphs represent the mean ± SEM of at least three independent experiments. #Statistical differences (###*p* < 0.005) compared to the HaCaT control cells not treated with DBE or H_2_O_2_. *Statistical differences (*p < 0.05, **p < 0.01, ***p < 0.005) compared to cells treated with H_2_O_2_.

Bax, a pro-apoptotic protein, and Bcl-2, an anti-apoptotic protein, are key regulators of cell survival and programmed cell death [26]. To assess their involvement in DBE-mediated protection, we measured their protein expression levels (Fig. 3A). In HaCaT keratinocytes exposed to H_2_O_2_, DBE did not restore Bcl-2 expression. However, pretreatment with 50-80 μg/ml DBE effectively suppressed the H_2_O_2_-triggered upregulation of Bax, such that its levels were closer to those observed in control cells (Fig. 3B and 3C). Furthermore, analysis of the Bax/Bcl-2 ratio revealed a dose-dependent reduction following DBE treatment. These findings suggest that DBE mitigates H_2_O_2_-induced apoptosis in HaCaT keratinocytes by modulating the balance between pro- and anti-apoptotic signaling.

### DBE Inhibited MAPK Signaling Pathway in H_2_O_2_-Induced HaCaT Keratinocytes

**Fig. 4:**
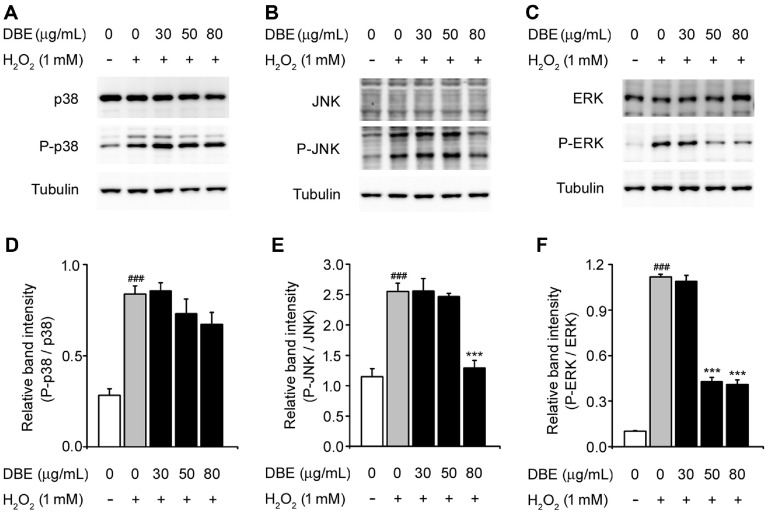
Effect of DBE on the expression of MAPK proteins in HaCaT keratinocytes exposed to hydrogen peroxide. (**A**) Western blot bands corresponding to p38 and P-p38 in HaCaT keratinocytes, which were pre-treated with DBE and then exposed to H_2_O_2_. Tubulin was used as a loading control. (**B**) Western blotting bands corresponding to JNK and p-JNK. (**C**) Western blotting bands corresponding to ERK and p-ERK. (**D**) Relative band intensity of P-p38 was quantified using densitometric analysis and normalized to that of p38. (**E**) Relative band intensity of p-JNK was quantified using densitometric analysis and normalized to that of JNK. (**F**) Relative band intensity of p-ERK was quantified using densitometric analysis and normalized to that of ERK. The values in the bar graphs represent the mean ± SEM of at least three independent experiments. # Statistical differences (###*p* < 0.005) compared to the HaCaT control cells not treated with DBE or H_2_O_2_. *Statistical differences (*p < 0.05, **p < 0.01, ***p < 0.005) compared to cells treated with H_2_O_2_.

The MAPK pathway, which includes p38, JNK, and ERK, is abnormally activated by environmental stress, playing key roles in immune responses, inflammation, and apoptosis [27, 28]. To better understand the molecular mechanisms by which DBE suppresses H_2_O_2_-associated cellular damage, we investigated the MAPK signaling pathway. Cells exposed to H_2_O_2_ showed increased levels of phosphorylated p38, JNK, and ERK compared with untreated cells. DBE treatment reduced JNK and ERK phosphorylation in a dose-dependent manner. However, the phosphorylation levels of p38 remained unaffected (Fig. 4B and 4C). These observations indicated that DBE protects HaCaT keratinocytes against oxidative stress by inhibiting the activation of MAPK (JNK and ERK) signaling.

### DBE Attenuated H_2_O_2_-Stimulated NF-κB p65 Phosphorylation and COX-2 Expression

**Fig. 5:**
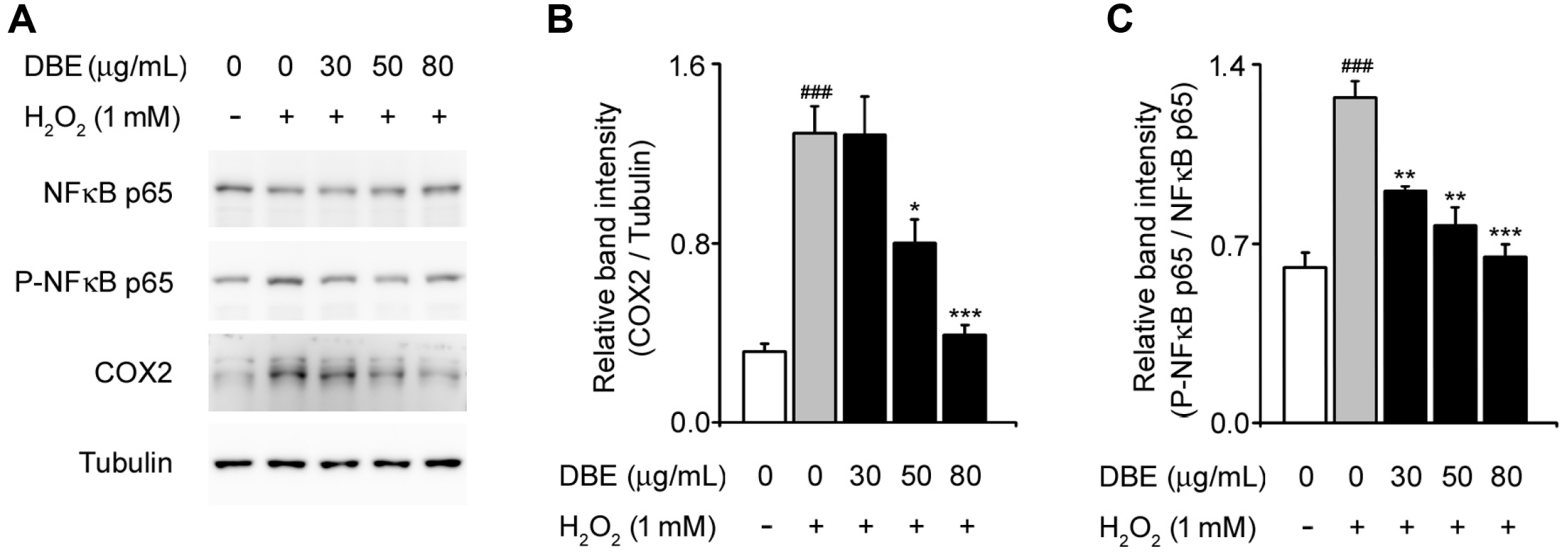
Effect of DBE on the phosphorylation of NF-κB and COX-2 in HaCaT keratinocytes exposed to hydrogen peroxide. (**A**) Western blot bands corresponding to NF-κB p65, P-NF-κB p65, and COX2 in HaCaT keratinocytes were pre-treated with DBE and then exposed to H_2_O_2_. (**B**) Relative band intensity of COX2 was quantified using densitometric analysis and normalized to that of tubulin. (**C**) Relative band intensity of P-NF-κB p65 was quantified using densitometric analysis and normalized to that of NF-κB p65. #Statistical differences (###*p* < 0.005) compared to the HaCaT control cells not treated with DBE or H_2_O_2_. The values in the bar graphs represent the mean ± SEM of at least three independent experiments. # Statistical differences (###*p* < 0.005) compared to the HaCaT control cells not treated with DBE or H_2_O_2_. *Statistical differences (*p < 0.05, **p < 0.01, ***p < 0.005) compared to cells treated with H_2_O_2_.

Next, we analyzed the effects of DBE on the expression of inflammation-associated proteins to elucidate its protective mechanisms. Nuclear factor-κB (NF-κB), a key transcription factor, plays a central role in inflammatory responses triggered by oxidative stress [29, 30]. Therefore, we assessed protein levels of NF-κB p65, phosphorylated NF-κB p65, and COX-2 in HaCaT keratinocytes under H_2_O_2_-induced oxidative stress [31]. Compared to the untreated controls, H_2_O_2_ treatment significantly increased phosphorylated NF-κB p65 and COX-2 expression (Fig. 5A) However, DBE administration reduced NF-κB p65 phosphorylation in a dose-dependent manner (Fig. 5B) and suppressed H_2_O_2_-induced COX-2 upregulation (Fig. 5C). Notably, the total NF-κB p65 protein levels remained unchanged regardless of H_2_O_2_ exposure or DBE treatment. These findings suggest that DBE may protect HaCaT keratinocytes from oxidative stress by modulating NF-κB-mediated inflammatory signaling.

## Discussion

Oxidative stress is a critical factor in the pathogenesis of various dermatological conditions, including atopic dermatitis and psoriasis [32]. Among ROS, H_2_O_2_ plays a particularly significant role due to its ability to penetrate cell membranes, initiate lipid peroxidation, disrupt cellular homeostasis, and induce apoptosis [33-35]. In this study, we demonstrated that DBE confers protection against H_2_O_2_-induced oxidative stress in HaCaT keratinocytes.

To evaluate the safety of DBE, we assessed its effects on HaCaT cells, showing that a concentration of 100 μg/ml led to a marginal reduction in cell viability after 24 h of exposure, indicating potential cytotoxic effects at higher concentrations. Based on these results, we selected 30, 50, and 80 μg/ml as optimal concentrations for subsequent experiments. When HaCaT keratinocytes were exposed to H_2_O_2_, cell viability was reduced to approximately 51%. However, DBE restored cell viability in a concentration-dependent manner, suggesting its protective role against oxidative damage.

To investigate the protective effects of DBE, we examined its role in preventing apoptosis. H_2_O_2_-treated cells exhibited characteristic apoptotic features, including cellular shrinkage and detachment, both of which were significantly attenuated by DBE pretreatment. The Trypan blue exclusion assay confirmed that DBE enhanced cell viability under oxidative stress. At the molecular level, apoptosis was largely mediated by the activation of caspases, particularly caspase-3 [25, 36]. Western blotting revealed a substantial increase in cleaved caspase-3 expression following H_2_O_2_ exposure, whereas DBE treatment significantly reduced the cleaved caspase-3 levels in a dose-dependent manner. These findings suggest that DBE exerts protective effects by interfering with the apoptotic signaling pathways.

Bax, a pro-apoptotic factor, and Bcl-2, an anti-apoptotic protein, determine cell fate by modulating mitochondrial membrane integrity [26, 37]. Our results indicate that while DBE pretreatment did not restore Bcl-2 levels, it effectively suppressed H_2_O_2_-induced Bax upregulation, particularly at 50 and 80 μg/ml. Consequently, the Bax/Bcl-2 ratio decreased in a dose-dependent manner, further supporting the anti-apoptotic potential of DBE against oxidative stress-induced cell death [38].

To elucidate the molecular mechanisms underlying DBE-mediated cellular protection, we investigated its effect on MAPK signaling, which is a crucial regulator of cellular responses to oxidative stress [39]. H_2_O_2_ exposure significantly increased the phosphorylation of JNK, ERK, and p38 MAPK, indicating activation of stress-responsive pathways [40]. Notably, DBE pretreatment selectively attenuated JNK and ERK phosphorylation in a concentration-dependent manner, whereas p38 phosphorylation remained unchanged. These findings suggest that DBE exerts its protective effects by selectively modulating MAPK signaling components rather than broadly suppressing the entire pathway.

In addition to its effects on cell survival and apoptosis, we examined the effects of DBE on inflammation-related signaling pathways [41]. NF-κB, a key transcription factor, plays a central role in regulating inflammatory responses under oxidative stress conditions [30, 42]. In our study, H_2_O_2_ treatment significantly enhanced the phosphorylation of NF-κB p65 and increased COX-2 expression, which is a well-established marker of inflammation [43]. DBE pretreatment effectively reduced NF-κB p65 phosphorylation and downregulated COX-2 expression in a dose-dependent manner. Interestingly, total NF-κB p65 protein levels remained unaltered, suggesting that DBE modulates NF-κB activation rather than affecting its overall expression.

Taken together, this study provides evidence for the protective potential of DBE against oxidative stress-induced cellular damage in HaCaT keratinocytes. DBE promotes cell survival, suppresses apoptosis, and modulates key signaling pathways, including MAPK and NF-κB, which are central to oxidative stress responses. These findings establish a foundation for further investigation of DBE as a potential therapeutic or cosmetic agent for skin conditions linked to oxidative stress while providing insights into its possible clinical applications.
